# Sulfanyl Porphyrazines with Morpholinylethyl Periphery—Synthesis, Electrochemistry, and Photocatalytic Studies after Deposition on Titanium(IV) Oxide P25 Nanoparticles

**DOI:** 10.3390/molecules26082280

**Published:** 2021-04-15

**Authors:** Tomasz Koczorowski, Wojciech Szczolko, Anna Teubert, Tomasz Goslinski

**Affiliations:** 1 Chair and Department of Chemical Technology of Drugs, Poznan University of Medical Sciences, Grunwaldzka 6, 60-780 Poznan, Poland; wszczolko@wp.pl (W.S.); tomasz.goslinski@ump.edu.pl (T.G.); 2 Institute of Bioorganic Chemistry, Polish Academy of Sciences, Z. Noskowskiego 12, 61-704 Poznan, Poland; ateubert@ibch.poznan.pl

**Keywords:** catalysis, electrochemistry, morpholine, porphyrazine, titanium(IV) oxide

## Abstract

The syntheses, spectral UV–Vis, NMR, and electrochemical as well as photocatalytic properties of novel magnesium(II) and zinc(II) symmetrical sulfanyl porphyrazines with 2-(morpholin-4-yl)ethylsulfanyl peripheral substituents are presented. Both porphyrazine derivatives were synthesized in cyclotetramerization reactions and subsequently embedded on the surface of commercially available P25 titanium(IV) oxide nanoparticles. The obtained macrocyclic compounds were broadly characterized by ESI MS spectrometry, 1D and 2D NMR techniques, UV–Vis spectroscopy, and subjected to electrochemical studies. Both hybrid materials, consisting of porphyrazine derivatives embedded on the titanium(IV) oxide nanoparticles’ surface, were characterized in terms of particle size and distribution. Next, they were subjected to photocatalytic studies with 1,3-diphenylisobenzofuran, a known singlet oxygen quencher. The applicability of the obtained hybrid material consisting of titanium(IV) oxide P25 nanoparticles and magnesium(II) porphyrazine derivative was assessed in photocatalytic studies with selected active pharmaceutical ingredients, such as diclofenac sodium salt and ibuprofen.

## 1. Introduction

Porphyrazines (Pzs) are synthetic tetrapyrrole macrocyclic molecules, known as aza-analogues of porphyrins. Their physicochemical properties can be tuned by the exchange of the central metal cation or by peripheral substitution [1]. Substituted porphyrazines reveal high absorption in the UV–Vis region and good effectiveness for singlet oxygen generation. In addition, they are usually soluble in organic solvents [2,3,4,5]. Their unique physicochemical properties, including optical and electrochemical ones, make them useful in biosensing [6], photocatalysis [7], nonlinear optics [8], and biomedicine, where they can be considered as photosensitizers for photodynamic therapy [9]. Amino and sulfanyl porphyrazines can be obtained from a cyclotetramerization reaction starting from diaminomaleonitrile or dimercaptomaleonitrile disodium salt derivatives, respectively.

Porphyrazines peripherally substituted with sulfanyl moieties have been studied widely over the last twenty years. They are well-soluble in common organic solvents [10,11,12], and present interesting optical [13,14] and electrochemical properties, and have therefore been applied as sensing materials in technology [15,16,17,18,19,20,21]. Other applications of sulfanyl Pzs concern photodynamic therapy (PDT) [22,23,24], wastewater treatment [25,26,27], and catalysis [28,29,30,31]. Symmetrical octa-substituted sulfanyl porphyrazines, unlike their unsymmetrical derivatives, usually present low singlet oxygen generation quantum yields, and therefore their biological activities are limited [32,33]. Recently, many symmetrical and unsymmetrical magnesium(II) and zinc(II) sulfanyl porphyrazines with bulky dendrimeric periphery were synthesized and evaluated in terms of their suitability for photodynamic therapy (PDT) [34,35,36,37,38]. In addition, some sulfanyl porphyrazines and phthalocyanines with morpholinyl moieties were studied as photosensitizers for PDT and photodynamic antimicrobial chemotherapy (PACT) and revealed promising potential [39,40,41].

Due to the presence of an expanded aromatic system in porphyrazines, they are often highly hydrophobic, and therefore less soluble or even insoluble in water. Thus, diverse methods, using specific carriers, have been employed to allow porphyrazines and related compounds to form stable suspensions in aqueous solutions. The carriers most widely applied in medicine and technology are liposomes [24,42,43], metal and metal oxide nanoparticles [44,45,46], and polymeric nanomaterials [47]. Among the metal oxide nanoparticles, one of the most interesting is titanium(IV) oxide nanoparticles (TiO_2_), which have unique photochemical features. Uncoated TiO_2_ nanoparticles are photoactive only when irradiated with UV light. However, TiO_2_ nanocarriers coated with photoactive compounds, like porphyrinoid macrocycles, when irradiated with light of an appropriate energy, can absorb light and participate in energy transfer, and thus more effectively take part in photochemical reactions [48,49,50].

Herein, we present the synthesis, spectral UV–Vis, NMR, and electrochemical as well as photocatalytic properties of novel magnesium(II) and zinc(II) symmetrical sulfanyl porphyrazines with 2-(morpholin-4-yl)ethylsulfanyl peripheral substituents. The synthesized macrocyclic compounds were embedded on the surface of commercially available P25 titanium(IV) oxide nanoparticles. The obtained grafted hybrid material was subjected to photocatalytic studies with 1,3-diphenylisobenzofuran, a known singlet oxygen quencher, and with diclofenac sodium salt and ibuprofen as examples of active pharmaceutical ingredients.

## 2. Results and Discussion

### 2.1. Synthesis and Physicochemical Characterization

**Scheme 1:**
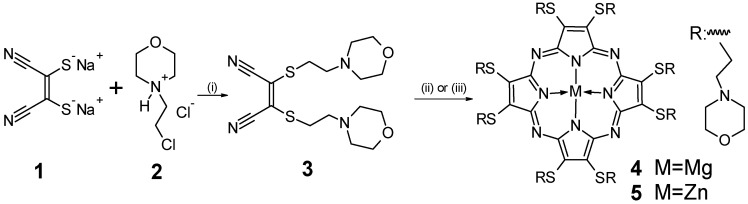
Reagents and conditions: (i) K_2_CO_3_, DMF, 60 °C, 24 h; (ii) Mg(*n*-BuO)_2_, *n*-butanol, reflux, 24 h; (iii) Zn(OAc)_2_, DBU, *n*-pentanol, reflux, 24 h; DBU—1,8-diazabicyclo[5.4.0]undec-7-ene, DMF—dimethylformamide.

The synthetic pathway was based on the alkylation reaction of commercially available mercaptomaleonitrile disodium salt (**1**) with 4-(2-chloroethyl)morpholine hydrochloride (**2**) in dimethylformamide (DMF), and with potassium carbonate as a base, which led to compound **3** (Scheme 1) [20,51]. Next, the Linstead macrocyclization reaction of **3** with magnesium butanolate as a base in *n*-butanol led to novel symmetric sulfanyl magnesium(II) porphyrazine **4** [52]. Simultaneously, compound **3** was also used in the macrocyclization reaction with Zn(OAc)_2_ and 1,8-diazabicyclo[5.4.0]undec-7-ene (DBU) in *n*-pentanol to give zinc(II) porphyrazine **5** [53].

All synthesized novel macrocyclic compounds were characterized using various analytical techniques, including high-resolution mass spectra (ESI), one- and two-dimensional NMR spectroscopy, and UV–Vis spectrophotometry. Notably, Pzs **4** and **5** have very low melting points at 113–116 °C and 121–123 °C, respectively, which is relatively low compared with other sulfanyl and amino porphyrazines that melt over 300 °C [20,54].

**Figure 1:**
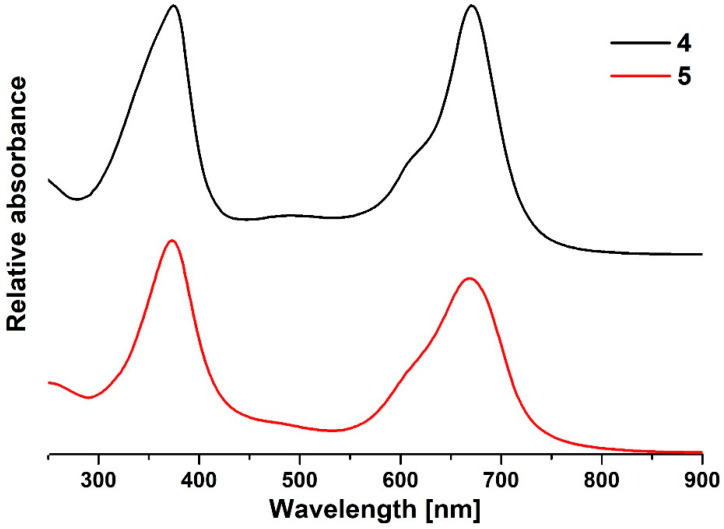
The UV–Vis spectrum of Pz **4** (black line) and Pz **5** (red line) in dichloromethane.

In the UV–Vis spectra of porphyrazines **4** and **5** in dichloromethane, two intensive bands were found: a Soret or B band in the range of 250–400 nm, and a Q band between 600 and 800 nm (Figure 1). Magnesium(II) Pz **4** revealed strong absorption Soret and Q bands with maxima at 375 m and 671 nm, whereas zinc(II) Pz **5** maxima appeared at 373 and 668 nm, respectively. The only difference between the UV–Vis spectra of **4** and **5** was noted in the region between 450 and 550 nm, where, for Pz **4,** only a weak absorption maximum at 497 nm appeared. A similar effect was noted before for sulfanyl porphyrazines with peripheral phthalimide motifs [38].

In the NMR spectra of porphyrazines **4** and **5**, which were recorded in pyridine-*d*_5_, four distinguishable signals in the aliphatic region were noted in the ^1^H NMR, whereas there were six signals in the ^13^C NMR (four aliphatic and two aromatic, originating from pyrrolyl rings of macrocycle, see Supplementary Materials). The two-dimensional technique ^1^H-^1^H COSY NMR was used to assist allocation and analysis of protons within ethylsulfanyl and morpholinyl substituents. Signal shift analyses performed for Pz **4** and Pz **5** indicated similarities for two morpholinyl proton signals and one of two methylene signals within the ethylene linker. A slight difference in the shifts of signals was noted for another methylene group within the ethylene linker. These methylene protons appeared as a singlet at 4.64 ppm for Pz **4**, and as a multiplet in the range of 3.81–3.89 ppm for Pz **5**. In addition, in the ^1^H NMR spectra of Pz **4** and **5**, the proton signals were generally up-field shifted in comparison with those in the structure of symmetrical magnesium(II) phthalocyanine with eight 2-(morpholin-4-yl)ethoxy substituents [39]. This finding could be explained by a higher electronegativity of oxygen atoms than sulfur present in the peripheries of both groups of macrocycles.

### 2.2. Electrochemistry

**Figure 2:**
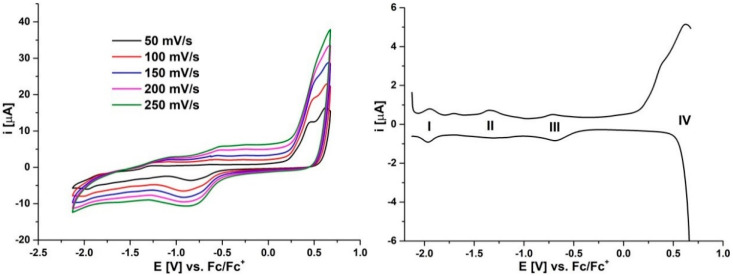
The cyclic and differential pulse voltammograms of porphyrazine **4** in 0.1 M TBAP/DCM. The DPV parameters: modulation amplitude 20 mV and step rate 5 mV·s^−1^.

**Figure 3:**
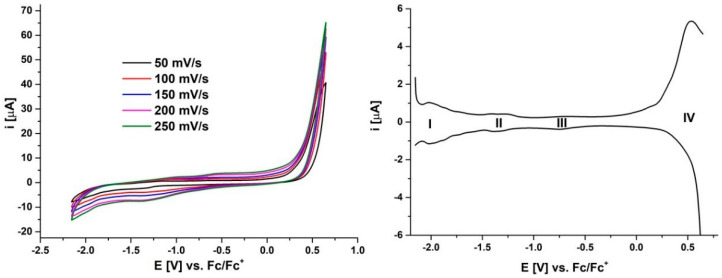
The cyclic and differential pulse voltammograms of porphyrazine **5** in 0.1 M TBAP/DCM. The DPV parameters: modulation amplitude 20 mV and step rate 5 mV·s^−1^.

The electrochemical study was performed to assess the electrochemical properties of the obtained porphyrazines, aiming to propose their prospective potential applicabilities. The cyclic (CV) and differential pulse (DPV) voltammetry measurements were conducted in organic solvent (dichloromethane), with the addition of supporting electrolyte: 0.1 M tetrabutylammonium perchlorate. The classic three-electrode system was employed with the glassy carbon working electrode. Due to the use of Ag wire as a pseudo-reference electrode, the ferrocene was added as an internal standard, and all results were adjusted to the ferrocene/ferrocenium peak potential. In the CV experiments, the scan potential range was set between 50 and 250 mV/s. The obtained results are presented in Figure 2 and Figure 3 and Table 1.

In the voltammograms recorded for both porphyrazines **4** and **5**, four redox peak potentials were noted. Almost all redox peaks observed in the CV voltammograms are irreversible due to the aggregation-disaggregation behavior of porphyrazines in dichloromethane; thus, redox pairs can be observed only in the DPV measurements. A similar phenomenon was previously observed for iron(II) porphyrazine bearing identical periphery to both herein studied Pzs [20]. However, the oxidation peaks (**IV**) at 0.62 V for Pz **4** and 0.53 V for Pz **5** were noted in the DPV voltammogram only when the applied potential was increasing over time from −2.0 to 0.7 V. Conversely, when the applied potential was decreasing over time, oxidation peaks were not observed (Figure 2 and Figure 3). The oxidation peak currents were at least five times higher than the highest reduction peak (**I**) in the case of both porphyrazines (Figure 2 and Figure 3). Such a high oxidation peak current could also be a result of an aggregation phenomenon, which was previously observed for other sulfanyl porphyrazines [15]. Notably, the peak potentials of zinc(II) porphyrazine **5** shifted to more negative potentials in comparison with magnesium(II) complex **4** (Table 1), which is the result of different metal cations present inside their cores [55].

Measurements performed for amino porphyrazines in dichloromethane indicated that the first oxidation peak appears below 0 V vs. Fc/Fc^+^ [56,57]. A different situation was observed when alkyl- or phenylsulfanyl substituents were present in the macrocyclic periphery. Then, peaks shifted toward positive values due to a strong electron-withdrawing effect [55,58]. The oxidation peak potentials (IV) of Pzs **4** and **5** comply with this rule.

In order to calculate the HOMO-LUMO energy levels in compounds such as porphyrinoid macrocycles, electrochemical measurements were used. The electrochemical energy gap (*E*_gap el_) was calculated by determining the onset potentials of first oxidation and first reduction processes originating from the porphyrazine ring with the use of the following equations:

*E*_HOMO_ = −(*V*_onset ox_ − *V*_FOC_ + 4.8) eV,

*E*_LUMO_ = −(*V*_onset red_ − *V*_FOC_ + 4.8) eV,

*E*_gap el_ = (*E*_LUMO_ − *E*_HOMO_) eV

**Figure 4:**
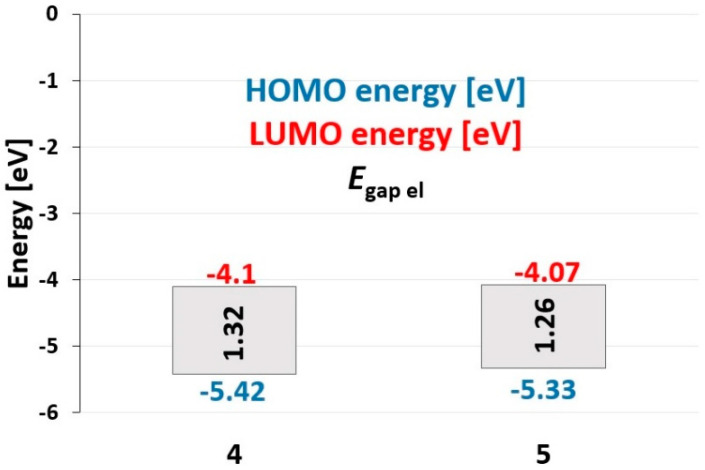
The HOMO-LUMO energy levels estimated for electrochemical data for porphyrazines **4** and **5**.

In the above equations, *V*_FOC_ stands for the ferrocene half-wave potential, *V*_onset ox_ is assigned as the Pz oxidation onset, and *V*_onset red_ represents the Pz reduction onset. For all these values (eV), the calculations of the first oxidation and the first reduction were adjusted to the ferrocene’s energy level at −4.8 eV. The value of 4.8 eV refers to a standard electrode potential for normal hydrogen electrode (NHE) at −4.6 eV on the zero vacuum level scale, and a value of 0.2 eV versus NHE for the potential of ferrocene standard [59,60]. The calculated electrochemical energy gap was slightly higher for magnesium(II) porphyrazine **4** than for zinc(II) complex **5** (Figure 4 and Table 2).

At the same time as the previous calculations, we calculated the optical band gaps (*E*_gap opt_) based on the UV–Vis spectra Q band onsets, according to the equation E = *h*c/λ_onset_ [61]. Both electrochemical and optical energy gaps, which we obtained, were subsequently compared and are presented in Table 2. The optical band gaps (*E*_gap opt_) for porphyrazines **4** and **5** were found to be in agreement with those obtained from electrochemical measurements within approx. 0.2 eV.

### 2.3. TiO_2_ Deposition and Characterization

Titanium(IV) oxide (TiO_2_) nanoparticles have often been utilized in diverse studies as carriers for photosensitizers [62,63]. Among many titanium(IV) oxide types, the commercially available P25, consisting of a mixture of crystal phases of anatase and rutile, is the most popular. Herein, the hybrid materials, type Pz@P25, were prepared by depositing porphyrazine **4** or **5** on the surface of TiO_2_ nanoparticles. The solutions of macrocycles were added to titania suspension, sonicated, and mixed for 72 h, yielding **4**@P25 and **5**@P25, respectively. The resulting hybrid materials contained 5% (*w*/*w*) of the macrocycle. The sizes and the dispersities of the obtained nanomaterials were subjected to the detailed analyses using a NanoSight LM10 instrument (sCMOS camera, 405 nm laser), equipped with a nanoparticle tracking analysis system. The diameters of the obtained hybrid materials were assessed and compared with the pure TiO_2_ nanoparticles. The results are presented in Table 3.

Considering the measured particle size values, strong agglomeration of the hybrid nanoparticles was observed. The mean particle sizes of the **4**@P25 and **5**@P25 were four times higher than the unmodified P25 (74.8 ± 7.7 nm). This could suggest that the deposition of the macrocycles strongly influences the titanium(IV) oxide nanoparticles. In addition, in both cases, the calculated polydispersity indices were below 0.2, which indicated that the distributions of nanoparticles within the studied hybrid materials are monodisperse. It also seems that the presence of sulfanyl porphyrazines on the surface of P25 nanoparticles hampers the electrostatic interactions between Pz@P25 nanoparticles and allows obtaining Pz@P25 of specific diameters.

### 2.4. Photocatalysis

**Scheme 2:**
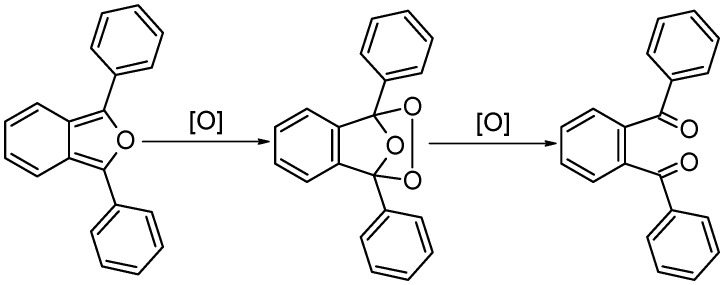
The oxidation of 1,3-diphenylisobenzofurane to 1,2-dibenzoylbenzene.

All hybrid materials were assessed for their photocatalytic oxidation abilities. These properties were studied with the use of a known singlet oxygen quencher, 1,3-diphenylisobenzofurane (DPBF), according to previously presented procedures [20,65]. In the UV–Vis spectrum, the decrease in the DPBF absorption band at 413 nm over a period of time results from the transformation of DPBF towards the new product, which is 1,2-dibenzoylbenzene (Scheme 2).

Measurements were performed in DMF at ambient temperature, and with the use of red-light LED lamps (665 nm). The intensity of light was adjusted to 10 mW/cm^2^. The irradiations were conducted in a 10 mm quartz cuvette equipped with a magnetic stirrer. The results of photocatalytic reactions were evaluated with the use of UV–Vis spectrophotometry.

**Figure 5:**
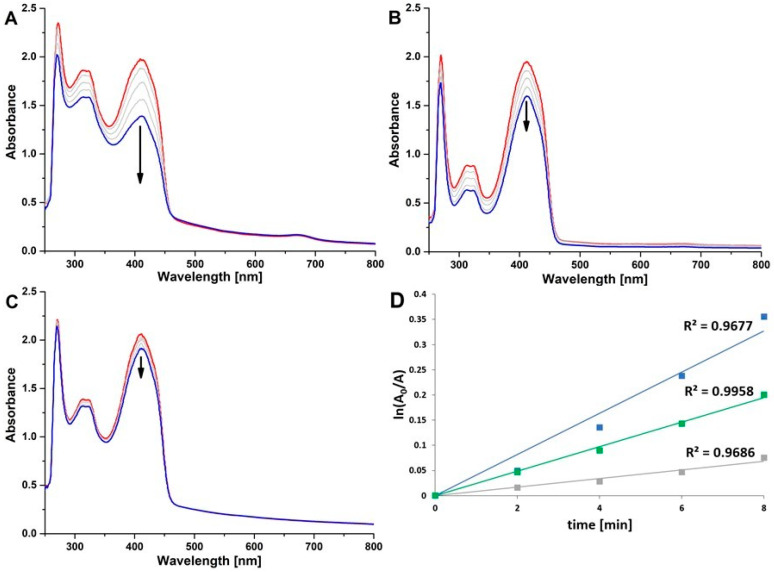
The UV–Vis spectra for the oxidation of DPBF in dimethylformamide in the presence of **4**@P25 (**A**), **5**@P25 (**B**), and P25 (**C**) as catalysts over a period of time. (**D**) the plots of DPBF absorbance in time in the presence of **4**@P25 (blue), **5**@P25 (green), and P25 (grey); DPBF—1,3-diphenylisobenzofurane.

The photocatalytic oxidations of DPBF were performed using three types of materials: **4**@P25, **5**@P25, and unmodified P25. Their catalytic activity was assessed by recording the UV–Vis scans within the range of 250–800 nm for 8 min, and every 2 min. The **4**@P25 hybrid material containing magnesium(II) sulfanyl porphyrazine deposited on the surface of TiO_2_ nanoparticles revealed the highest photocatalytic activity. Moderate activity was noted for **5**@P25 and the lowest activity for the unmodified P25 (Figure 5). In the case of **5**@P25 nanoparticles, the linear plots of DPBF absorbance were decreasing with time, which indicates that the photooxidation process follows the first-order kinetics (Figure 5D). In the parallel study, the R^2^ value measured for **4**@P25 hybrid material and P25 nanoparticles slightly deviated from unity.

**Figure 6:**
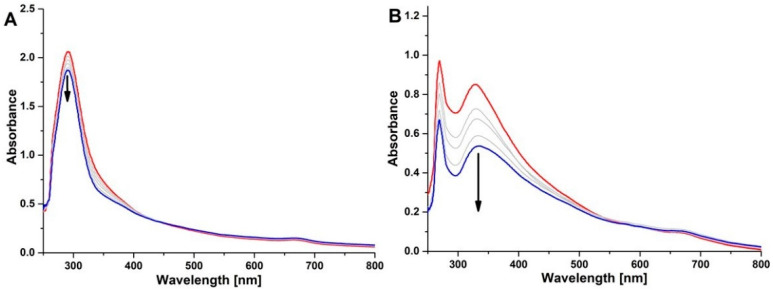
The UV–Vis spectra of photooxidation of diclofenac sodium salt (**A**) and ibuprofen (**B**) in dimethylformamide with **4**@P25 as a catalyst over a period of time.

The results obtained in the photocatalytic oxidation study with DPBF indicated the **4**@P25 hybrid material as a candidate for further photocatalytic study with selected active pharmaceutical ingredients (APIs): diclofenac sodium salt and ibuprofen. Both APIs are common non-steroidal anti-inflammatory drugs, and therefore constitute an important component of drug-related pollutants in water. What is essential is that the UV–Vis method can be employed for the photodegradation study of both APIs. The photodegradation study was performed in the same conditions as previously established for the photooxidation of DPBF. The results are presented in Figure 6. In the UV–Vis spectra, decreases in both APIs absorbances over a period of time were observed, which indicates the photodegradation of the studied compounds. For this reason, the obtained hybrid material **4**@P25 can be considered an efficient heterogenic catalyst for further photooxidation studies of diverse organic compounds.

## 3. Materials and Methods

### 3.1. Materials and Instruments

All the reactions described in this paper were conducted under argon. Before attempting the reaction, the glassware was oven-dried (at 140 °C). All solvents were rotary evaporated under vacuum at or below 40 °C. All reaction temperatures reported in the experimental section refer to the external bath temperatures. The reactions were performed on a Heidolph MR Hei-Tec, equipped with Radleys Heat-On heating mantle. All solvents and reagents were obtained from commercial suppliers (Merck, Darmstadt, Germany; TCI, Zwijndrecht, Belgium, and Fluorochem, Hadfield, UK), and used without any further purification, unless otherwise stated. Melting points were measured with the use of a Stuart Bibby apparatus (Triad Scientific, Staffordshire, UK) and are uncorrected. Flash column chromatography was performed on a Merck neutral aluminum oxide gel, whereas thin-layer chromatography (TLC) was performed on aluminum oxide F254 plates (Merck) and visualized with a UV lamp (λ_max_ 254 or 365 nm). UV–Vis spectra were recorded with the use of an Ocean Optics USB 2000+ spectrometer (Ocean Opitics Inc., Largo, FL, USA). ^1^H NMR and ^13^C NMR spectra were recorded using Bruker Avance 400 and 500 (Bruker, Karlsruhe, Germany) spectrometers. Chemical shifts (δ) are specified in parts per million (ppm) and are referenced against a residual solvent peak (pyridine-*d*_5_), whereas coupling constants (*J*) are calculated in Hertz (Hz). The abbreviations *s*, *t*, and *m* refer to singlet, triplet, and multiplet, respectively. Mass spectra (ESI MS) were performed in the Wielkopolska Centre of Advanced Technologies, Adam Mickiewicz University in Poznan, Poland.

### 3.2. Synthesis

**2,3-Bis[2-(morpholin-4-ylo)ethylsulfanyl]-(2*Z*)-butene-1,4-dinitrile (3)** is a known compound synthesized and characterized earlier in our group [20]: dimercaptomaleonitrile disodium salt (558 mg; 3.0 mmol) (**1**), 4-(2-chloroethyl)morpholine hydrochloride (1.396 g; 7.5 mmol) (**2**) and K_2_CO_3_ (4.140 g; 30.0 mmol) were mixed in DMF (30 mL) at 60 °C for 24 h under argon. Next, the reaction mixture was cooled to room temperature and filtered through Celite, then the filtrate was evaporated with toluene to a dry solid residue. Crude solid was subjected to a flash column chromatography with Al_2_O_3_ (DCM:CH_3_OH; 50:1) to give **3** as yellow crystals (810 mg; 71% yield).

**{2,3,7,8,12,13,17,18-Octakis[2-(morpholin-4-yl)ethylsulfanyl]porphyrazinato} magnesium(II) (4)**: magnesium turnings (45 mg; 1.88 mmol) and iodide (1 crystal) were suspended in *n*-butanol (15 mL) and refluxed for 6 h in an inert atmosphere. After the reaction mixture was cooled to room temperature, compound **3** (692 mg; 1.88 mmol) was added and the mixture was refluxed for 18 h. Next the mixture was filtrated through Celite and the solvents were evaporated with toluene to a dry solid. Crude product was purified by column chromatography on Al_2_O_3_ (DCM:MeOH, 50:1) to give porphyrazine **4** (110 mg; 16% yield) as a green-blue solid: mp 113-116 °C; R*_f_* (DCM:MeOH:N(C_2_H_5_)_3_, 10:1:0.1) 0.42. UV–Vis (DCM): λ_max_, nm (logε) 375 (4.68), 497 (3.91), 671 (4.71). ^1^H NMR (400 MHz; pyridine-*d*_5_): δ*H*, ppm 2.60 (*s*, 32H, morph-CH_2_), 3.11 (*s*, 16H, CH_2_), 3.67 (*s*, 32H, morph-CH_2_), 4.64 (*t*, ^3^*J* = 10.0 Hz, 16H, CH_2_). ^13^C NMR (100 MHz; pyridine-*d*_5_): δ*C*, ppm 33.5; 54.5; 60.0; 67.5; 141.9; 158.4. HRMS ESI (pos): calc. for C_64_H_97_N_16_O_8_S_8_Mg *m*/*z* 1497.5291 [M + H]^+^; found *m*/*z* 1497.5340 [M + H]^+^.

**{2,3,7,8,12,13,17,18-Octakis[2-(morpholin-4-yl)ethylsulfanyl]porphyrazinato} zinc(II) (5)**: dimercaptomaleonitrile **3** (700 mg; 1.9 mmol), Zn(OAc)_2_ (175 mg, 0.95 mmol) and DBU (142 μL, 0.95 mmol) in *n*-pentanol (4 mL) were refluxed in an inert atmosphere for 18 h. Next, the reaction mixture was filtrated through Celite and the filtrate was evaporated with toluene to dryness. Crude solid was purified by column chromatography with Al_2_O_3_ (DCM:MeOH; 50:1→10:1) to give porphyrazine **5** (90 mg; 12%) as a dark green solid: mp 121--123 °C; R*_f_* (DCM:MeOH:N(C_2_H_5_)_3_, 10:1:0.1) 0.57. UV–Vis (DCM): λ_max_, nm (logε) 373 (4.79); 668 (4.71). ^1^H NMR (500 MHz; pyridine-*d*_5_): δ*H*, ppm 2.54--2.63 (*m*, 32H, morph-CH_2_), 3.09 (*s*, 16H, CH_2_), 3.63--3.69 (*m*, 32H, morph-CH_2_), 3.81–3.89 (*m*, 16H, CH_2_). ^13^C NMR (125 MHz; pyridine-*d*_5_): δ*C*, ppm 33.34; 54.37; 59.88; 67.38; 141.92; 157.34. HRMS ESI (pos): calc. for C_64_H_97_N_16_O_8_S_8_Zn *m*/*z* 1539.4721 [M+H]^+^; found *m*/*z* 1539.4756 [M + H]^+^.

### 3.3. Electrochemical Studies

The electrochemical studies were performed with a Metrohm Autolab PGSTAT128N potentiostat (Metrohm, Herisau, Switzerland). The data acquisition and storage were driven by Metrohm Nova 2.1.4 software (Metrohm). The measurements were obtained with the use of a glassy carbon (GC) working electrode (area = 0.071 cm^2^), Ag wire (pseudo-reference electrode), and a platinum wire (counter electrode). Before each procedure, the GC electrode was polished with aqueous 50 nm Al_2_O_3_ slurry (purchased from Sigma-Aldrich) using a polishing cloth and was subsequently washed in an ultrasonic bath with deionized water for 10 min to remove inorganic impurities. Ferrocene/ferrocenium couple (Fc/Fc^+^) was applied as an internal standard. The solvent (dichloromethane) containing a supporting electrolyte (0.1 M tetrabutylammonium perchlorate (TBAP)) in a glass cell (volume 10 mL) was deoxygenated by purging nitrogen gas for 10 min prior to each experiment. All electrochemical experiments were carried out at 22 °C. The solvent and reagent were purchased from Sigma-Aldrich Chemie GmbH, Steinheim, Germany.

### 3.4. Deposition of Porphyrazines on TiO_2_ P25 Nanoparticles

Studied porphyrazines were deposited on P25 Aeroxide^®®^ titanium(IV) oxide (TiO_2_) nanoparticles using the chemical deposition method [50]. In general, porphyrazine **4** or **5** in the amount of 5 mg was added to a dispersion of 100 mg P25 nanoparticles (sized approx. 21 nm) in 20 mL of dichloromethane:methanol mixture (1:1, *v*/*v*). After the reaction mixture had been stirred for 72 h, the solvents were evaporated on a rotary evaporator. Next, the obtained hybrid material was air dried for 24 h. The ratio of the macrocycle to the P25 TiO_2_ was 1:20 (*w*/*w*).

The hybrid materials were subjected to nanoparticle size measurements using a Malvern Panalytical NanoSight LM10 instrument (Malvern, UK), equipped with sCMOS camera, and 405 nm laser. The data acquisition and storage were provided by Nanoparticle Tracking Analysis (NTA) 3.2 Dev Build 3.2.16 software (Malvern, UK). Throughout, the nanoparticles’ dispersions were diluted with water (1 mg in 1 mL) to obtain the operating range of nanoparticle concentration. The measurements were performed at 25.0 ± 0.1 °C, and at the syringe pump infusion rate set to 100 µL/min.

### 3.5. Photocatalytic Studies

The photocatalytic studies were performed using a red-light LED lamp (EcoEnergy, Gdańsk, Poland) at wavelength 665 nm, and a power adjusted to 10 mW/cm^2^ with the use of an Optel radiometer. The measurements were conducted in a 10 mm quartz cuvette in *N*,*N*′-dimethylformamide (DMF). In the experiments with a reference standard 1,3-diphenylisobenzofurane (DPBF), 1 mL of 0.1 mM DPBF solution in DMF was mixed with 1 mL of TiO_2_ dispersion in DMF (0.1 mg/mL). In the experiments with active pharmaceutical ingredients (diclofenac sodium salt and ibuprofen), DPBF was replaced by 1 mL of 0.3 mM diclofenac sodium salt solution in DMF or 1 mL of 1.5 mM ibuprofen solution in DMF. The irradiations of mixtures were performed with an LED lamp within 8 min. The UV–Vis spectra were recorded every 2 min on an Ocean Optics USB 2000+ spectrometer (Ocean Optics Inc., Largo, FL, USA).

## 4. Conclusions

Two novel sulfanyl magnesium(II) and zinc(II) porphyrazines with morpholinylethyl periphery were synthesized in the cyclotetramerization reaction using a dimercaptomaleonitrile derivative. The obtained macrocyclic compounds were broadly characterized by ESI MS spectrometry, 1D and 2D NMR techniques, and UV–Vis spectroscopy. Both porphyrazines were subjected to electrochemical studies. Subsequently, the obtained porphyrazines were embedded on titanium(IV) oxide nanoparticles’ surface and characterized in terms of particle size and distribution. The obtained hybrid materials’ applicability was assessed in photocatalytic studies with a singlet oxygen quencher (DPBF) and selected drug active pharmaceutical ingredients (diclofenac sodium salt and ibuprofen). In the UV–Vis and NMR studies, the characteristic features of porphyrazines were confirmed. The electrochemical studies revealed four irreversible redox processes for both porphyrazines. In addition, the calculated electrochemical band gap values were found to be in agreement with the optical ones. Interestingly, the obtained hybrid materials presented four times higher particle sizes compared with unmodified titanium(IV) oxide P25 nanoparticles and were monodispersive. The **4**@P25 material was found to be the most active in comparative photocatalytic tests with 1,3-diphenylisobenzofurane, and it was therefore used in the photooxidation studies of diclofenac sodium salt and ibuprofen. The **4**@P25 material revealed good photocatalytic potential. For this reason, it can be considered in future photocatalytic experiments with various organic compounds and active pharmaceutical ingredients as a potential hybrid material for the photodegradation of various organic pollutants.

## Figures and Tables

**Table 1:** The electrochemical data of porphyrazines **4** and **5**.

Pz		MPz^(−2)^/ MPz^(−3)^ I	MPz^(−1)^/ MPz^(−2)^ II	MPz^(0)^/ MPz^(−1)^ III	MPz^(0)^/ MPz^(+1)^ IV
**4**	E_1/2_ [V] vs. Fc/Fc^+^	−1.95	−1.33	−0.70	0.62
**5**	E_1/2_ [V] vs. Fc/Fc^+^	−2.02	−1.34	−0.74	0.53

**Table 2:** The optical and electrochemical HOMO-LUMO band gaps for porphyrazines **4** and **5**.

	λ_max_ [nm]		λ_onset_ [nm]	Optical Band Gap *E*_gap opt_ [eV]	Electrochemical Band Gap *E*_gap el_ [eV]
	Soret	Q Band			
**4**	375	671	820	1.51	1.32
**5**	373	668	850	1.46	1.26

**Table 3:** The particle size distribution of P25, **4**@P25, and **5**@P25.

Material	Measured Particle Size (nm)	Polydispersity Index ^a^
P25	74.8 ± 7.7	0.17
**4**@P25	327.4 ± 15.5	0.13
**5**@P25	322.5 ± 28.2	0.15

^a^ Calculated according to the formula PDI = (SD/mean diameter)^2^ [64].

## Data Availability

Not applicable.

## Supplementary Materials

The following are available online: Figure S1. ^1^H NMR spectrum of **4** in pyridine-*d*_5_. # indicates solvent residual peaks. Figure S2. ^13^C NMR spectrum of **4** in pyridine-*d*_5_. # indicates solvent residual peaks. Figure S3. ^1^H-^1^H COSY NMR spectrum of **4** in pyridine-*d*_5_. Figure S4. ^1^H NMR spectrum of **5** in pyridine-*d*_5_. * indicates solvent residual peaks and # stands for water residual. Figure S5. ^13^C NMR spectrum of **5** in pyridine-*d*_5_. * indicates solvent residual peaks. Figure S6. ^1^H-^1^H COSY NMR spectrum of **5** in pyridine-*d*_5_.

## Sample Availability

Samples of the compounds **3**, **4**, and **5** are available from the authors.
