# Supplementary material for: Sulfanyl Porphyrazines with Morpholinylethyl Periphery—Synthesis, Electrochemistry, and Photocatalytic Studies after Deposition on Titanium(IV) Oxide P25 Nanoparticles
Source: Molecules. 2021 Apr 15;26(8):2280. doi: 10.3390/molecules26082280 (PMC8071117; doi:10.3390/molecules26082280)
Supplement: Supplementary file 1 [file molecules-26-02280-s001.zip › molecules-1182673-supplementary.pdf]

**Sulfanyl porphyrazines with morpholinylethyl periphery – synthesis, electrochemistry, and photocatalytic studies after deposition on titanium(IV) oxide P25 nanoparticles**

Tomasz Koczorowski[a,*], Wojciech Szczolko[a], Anna Teubert[b], Tomasz Goslinski[a]

[a] Department of Chemical Technology of Drugs, Poznan University of Medical Sciences, Grunwaldzka 6, 60-780 Poznan, Poland
[b] Institute of Bioorganic Chemistry, Polish Academy of Sciences, Z. Noskowskiego 12, 61-704 Poznan, Poland

[*] Correspondence: tkoczorowski@ump.edu.pl (TK)

# SUPPORTING INFORMATION

## Table of contents

**NMR studies:**

$^{1}$H NMR spectrum of **4** in pyridine-*d5*… ……………………………………………………2
$^{13}$C NMR spectrum of **4** in pyridine-*d5*……………………………………………………...2
$^{1}$H-$^{1}$H COSY NMR spectrum of **4** in pyridine-*d5*…………………………………………...3
$^{1}$H NMR spectrum of **5** in pyridine-*d5*……………………………………………………….4
$^{13}$C NMR spectrum of **5** in pyridine-*d5*……………………………………………………...4
$^{1}$H-$^{1}$H COSY NMR spectrum of **5** in pyridine-*d5*…………………………………………...5

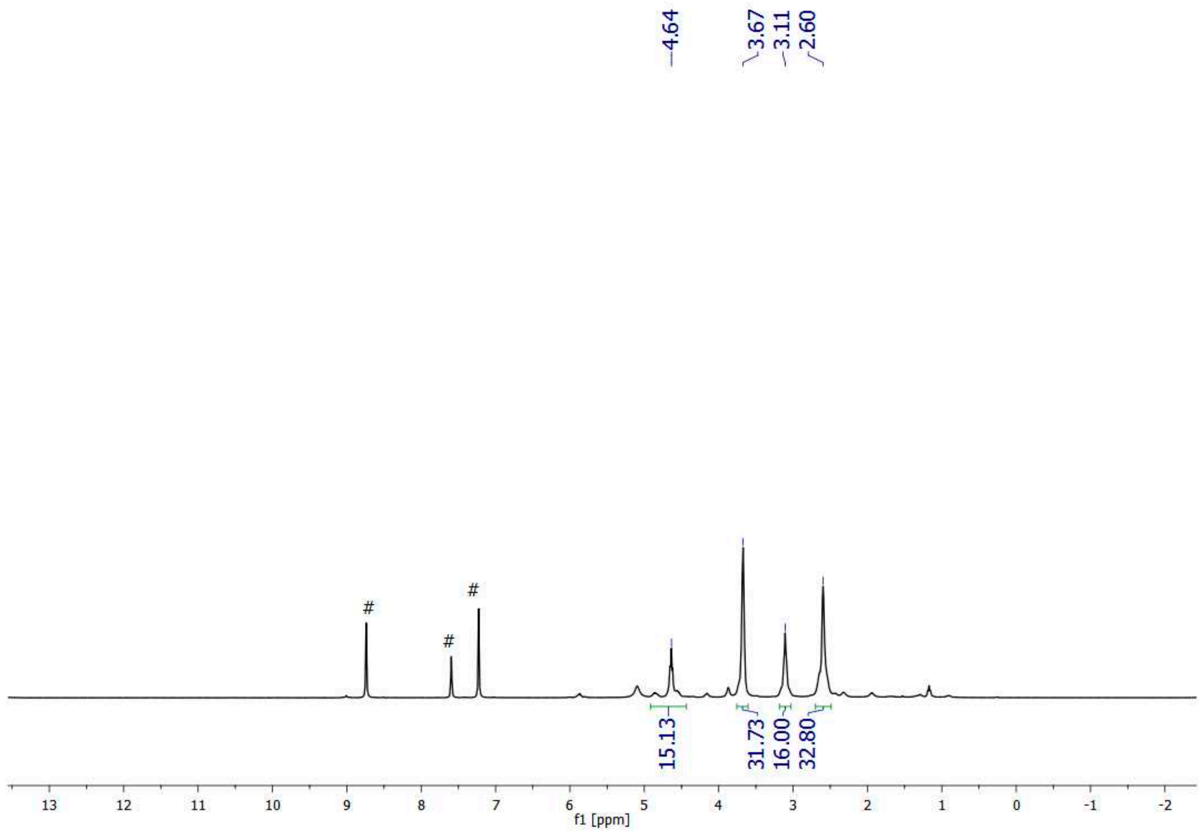


Figure S1. $^{1}$H NMR spectrum of **4** in pyridine-$d_5$. # indicates solvent residual peaks.

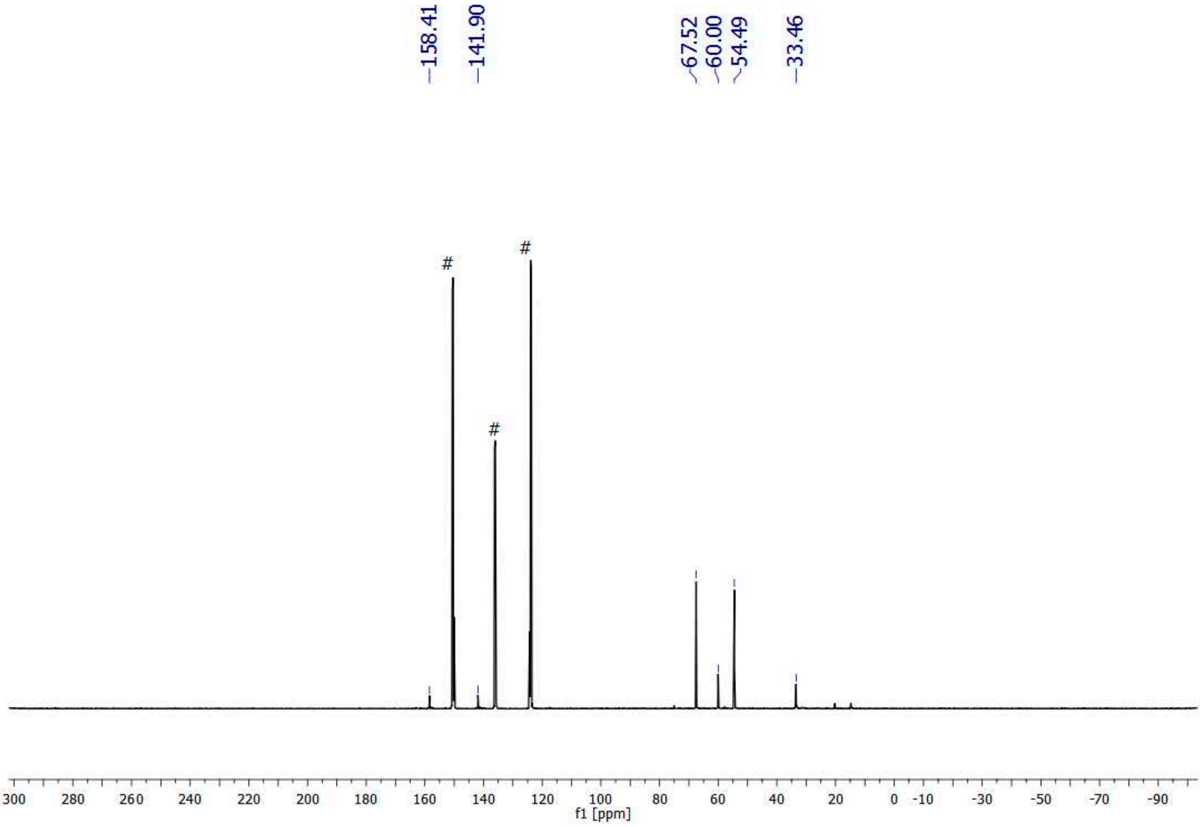


Figure S2. $^{13}$C NMR spectrum of **4** in pyridine-$d_5$. # indicates solvent residual peaks.

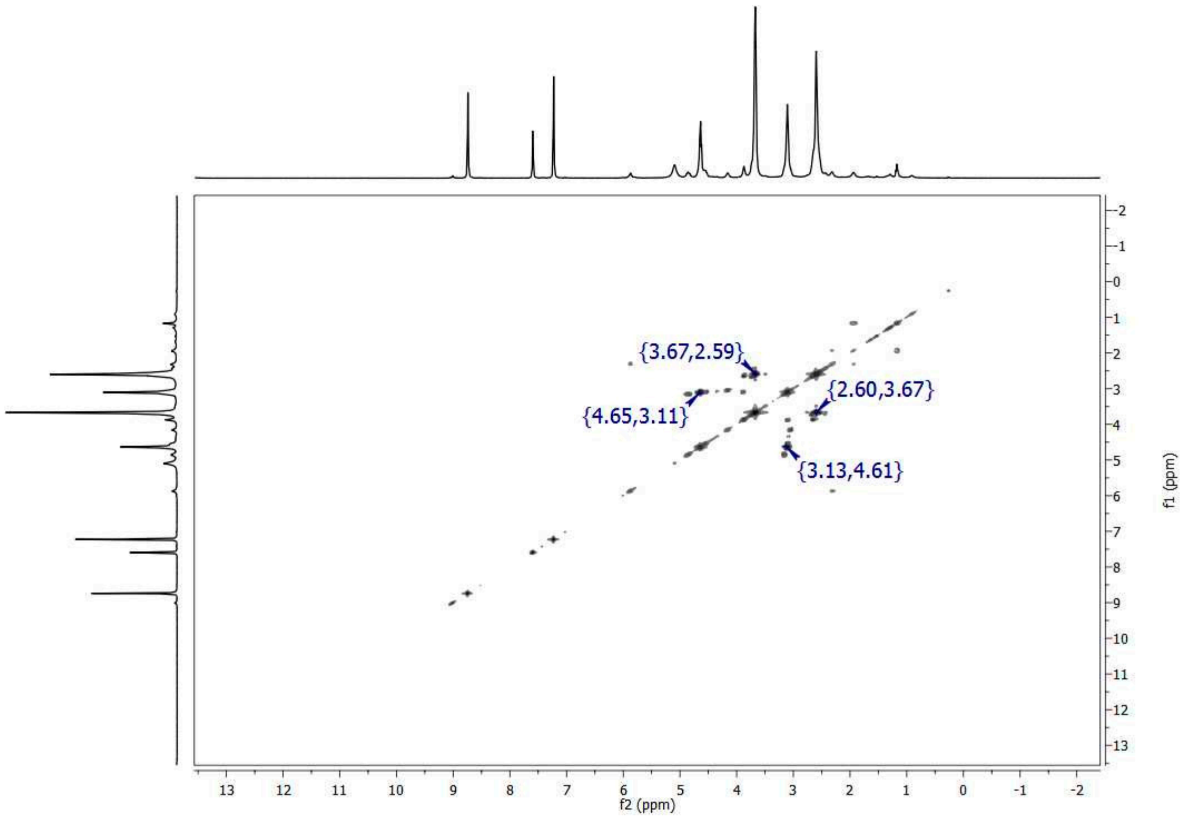


Figure S3. $^1$H-$^1$H COSY NMR spectrum of **4** in pyridine-$d_5$.

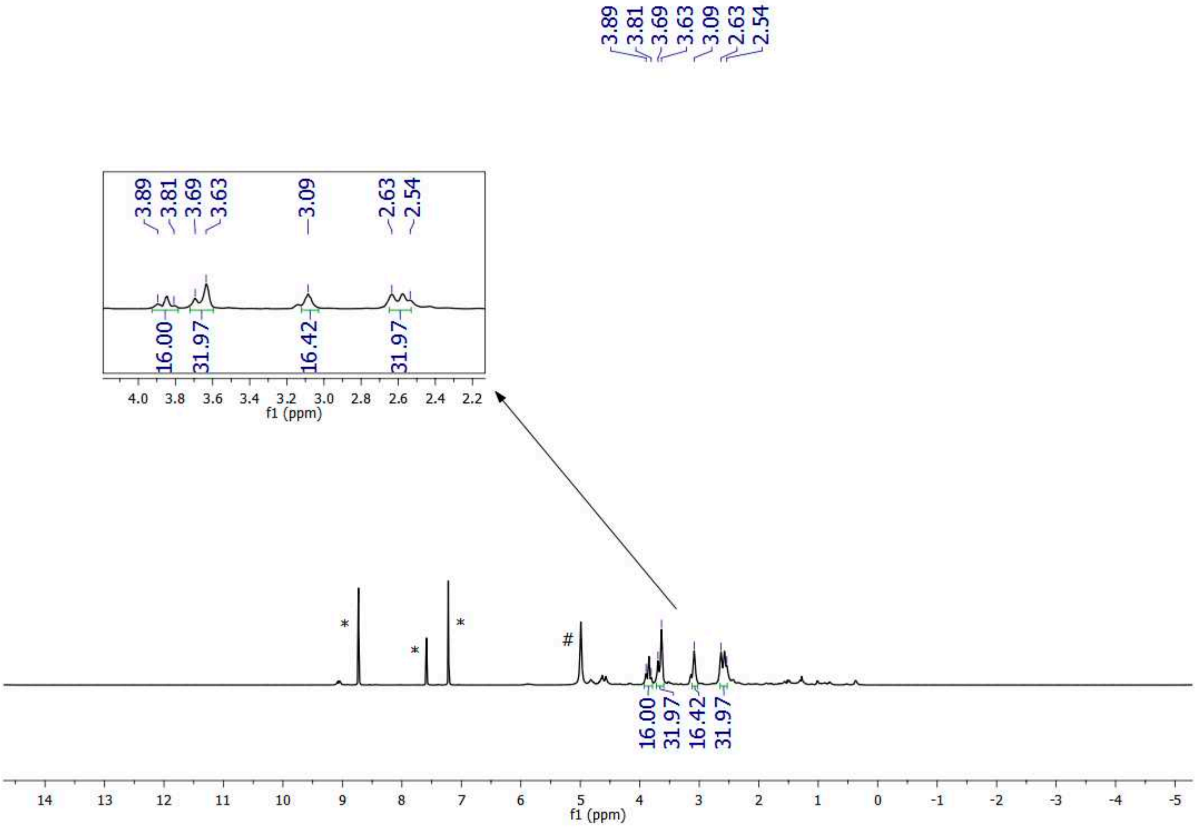


Figure S4. $^{1}$H NMR spectrum of **5** in pyridine-$d_5$. * indicates solvent residual peaks and # stands for water residual.

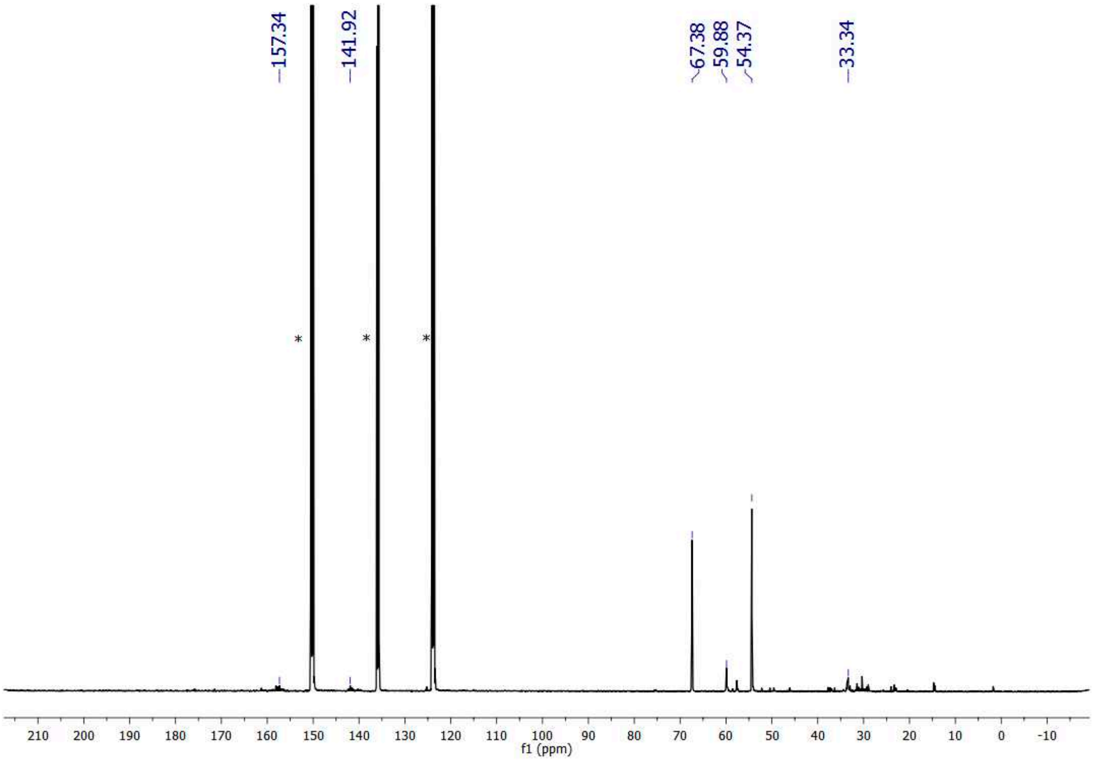


Figure S5. $^{13}$C NMR spectrum of **5** in pyridine-$d_5$. * indicates solvent residual peaks.

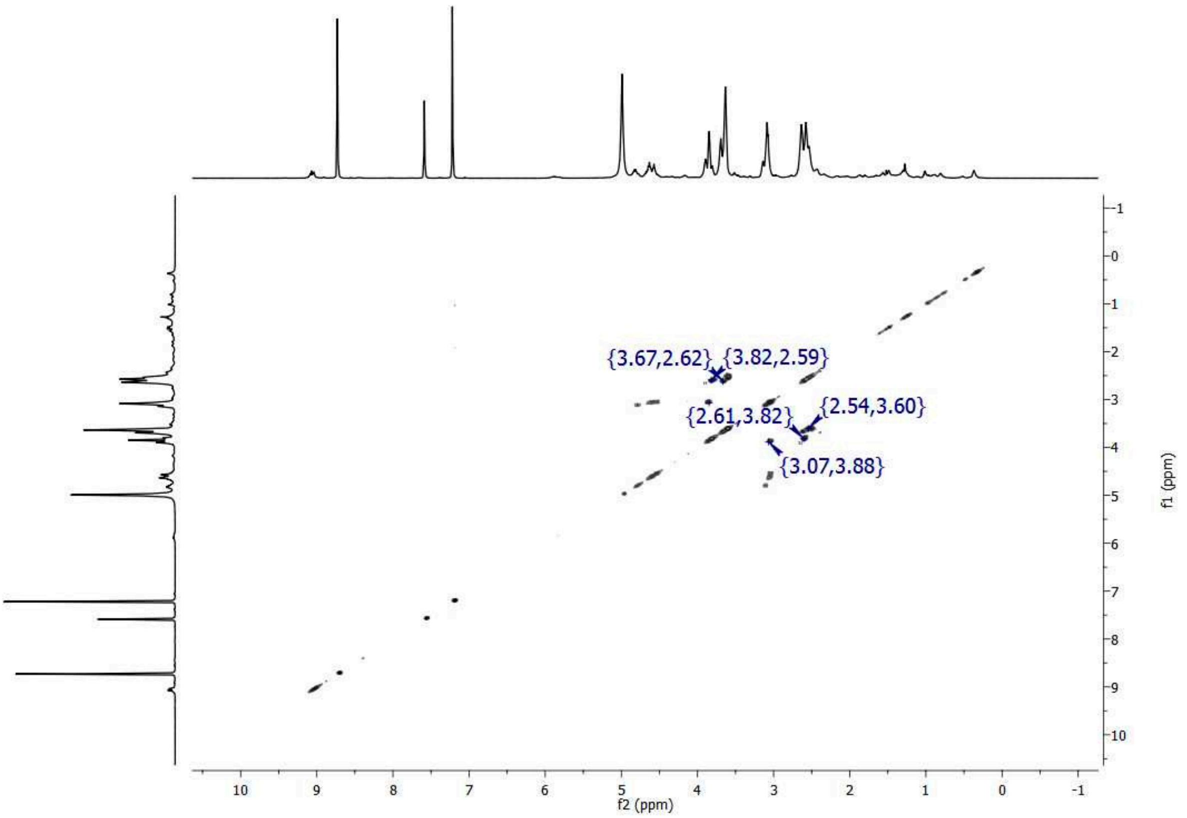


Figure S6. ¹H-¹H COSY NMR spectrum of **5** in pyridine-$d_5$.
